# Chitosan-Hyaluronate Hybrid Gel Intraarticular Injection Delays Osteoarthritis Progression and Reduces Pain in a Rat Meniscectomy Model as Compared to Saline and Hyaluronate Treatment

**DOI:** 10.1155/2012/979152

**Published:** 2012-05-07

**Authors:** Shachar Patchornik, Edward Ram, Noah Ben Shalom, Zvi Nevo, Dror Robinson

**Affiliations:** ^1^Chi2Gel Ltd., P.O. Box 633, 87516 Ofakim, Israel; ^2^Orthopedic Research Unit, Hasharon Hospital, Rabin Medical Center, Petah Tikva, Israel; ^3^Department of Genetics and Biochemistry, Sackler School of Medicine, Tel Aviv University, 69978 Tel Aviv, Israel

## Abstract

Chitosan-Hyaluronate hybrid gel (CHHG) is a self-forming thermo-responsive hydrogel. The current study was undertaken in order to assess the effect of CHHG on rat's surgically induced osteoarthritis. *Methods*. Thirteen rats were included in the study. In all rats weight-bearing was assessed using a Linton Incapacitance tester. All rats underwent bilateral medial partial meniscectomy. Four rats received a saline injection in the control knee and a 200-microliter injection of CHHG in the experimental knee. Five rats received a high-molecular weight hyaluronate injection to the control knee and a 200-microliter injection of CHHG in the experimental knee. Four rats underwent the same surgical procedure, allowed to recuperate for seven days and then CHHG and hyaluronate were injected. The animals were followed for 6 weeks. Two weeks after injection of a therapeutic substance the amount of weight-bearing on each knee was evaluated using a Linton Incapacitance meter. *Results*. Two weeks after induction of osteoarthritis there is less pain in the CHHG-treated knee than in the control-treated knee, as determined using a Lintron Incapacitance meter. After six-weeks the histological appearance of the CHHG-treated knee was superior to that of the controls. This is indicated by thicker cartilage remaining on the medial femoral condyle as well as less cyst formation in the CHHG-treated knee. *Discussion*. CHHG appears to delay progression of osteoarthritis and lessen pain in a rat surgically-induced knee osteoarthritis model. These results support other published results, indicating that there is an ameliorative effect of chitosan on human and rabbit osteoarthritis.

## 1. Introduction

Chitin is a nitrogen containing polysaccharide with mechanical strength and stability to chemical degradation. It is formed in the lower phyla of both the animal kingdom (Fauna) as the exoskeleton of invertebra like arthropodes, insects, crabs, lobsters, and mollusks, and in the plant kingdom (*Vegetative flora*) as well as in fungi. Chitin is probably the most common polymer found in animals, and can be hydrolyzed by a strong alkali to yield chitosan, a substance with quite different properties. Chitosan's unique features [1, 2] enable its use in various industries and medical applications.

In contrast to most other biopolymers, chitosan has a positive electrical charge due to amine groups, both free deacetylated and acetylated. This makes it elecrostatically attach to most living tissues that contain negatively charged surface matrices. Chitosan tends to support tissue healing by encouraging blood coagulation and allowing attachment of an endothelial layer on DeBakey-knitted grafts [1, 2]. Apparently the use of chitosan powder or pads allows rapid and scar free healing in many animal species including cats, dogs, cows, and zoo animals [3]. The improved healing might be related to increased permeability of cell membranes and is dependent on the presence of particles in the proper size [4].

Chitosan in the musculoskeletal system has a compound effect. Some studies reported diminished bone formation using chitosan scaffold as an interspace in a dog bone-distraction model [5] as compared with calcium phosphate. Intra-articular injection of chitosan is problematic as it has been shown to be very inflammatory [6]. However, once again the effect depends on the type of chitosan used. For example, Liu et al. have reported on the use of 2% carboxymethylated chitosan injected intra-articularly as a mitigator of osteoarthritis in a rabbit ACL-transection model [7], without observing an inflammatory effect, on the contrary the chitosan appeared to prevent metalloproteinase expression and protect the articular cartilage from osteoarthritic damage [7]. Indeed chitosan microspheres have been used as a slow-release agent for celecoxib injection into arthritic joints in rats [8]. The inflammatory effects of chitosan appear to be mediated via migration of polymorphonuclear cells to the particles [9]. The chitosan itself appears to induce osteopontin expression in white blood cells. Osteopontin is an inductor of attachment and spread of reparative cells [10]. In addition, it appears that chitosan induces collagen type II and aggrecan gene expression in a rabbit cartilage-injury model [11].

Thus, while intra-articular chitosan appears to prevent cartilage destruction and perhaps induce a reparative process due to cell attachment, it has also been associated with an inflammatory process which appears to be mitigated by cross-linking of the material and exposure of chitosan to autologous blood coagulation [12]. The application of chitosan appears to prevent adhesion formation in the rabbit knee following cartilage damage [13]. The current study has been performed in order to assess the potential beneficial effect of an in situ, cross-linked, and self-gelling chitosan-HA-hybrid formulation on the progression of knee osteoarthritis following meniscectomy in rabbits.

## 2. Methods

### 2.1. Animal Models and Procedures

Thirteen Wistar rats of 0.3-kilogram-weight male rats were used in the study. The study was approved by the Assaf Harofe Animal Ethical Committee. Knee osteoarthritis occurs predictably after partial medial meniscectomy [14]. The disease develops in a time-dependent and predictable fashion. It is a common model assessing the effect of antiosteoarthritis drugs.

General anesthesia was induced by Ketamine 80 mg/kg and Xylazine 8 mg/kg [15]. In the right knee, 200-microliters of 2% (w/v) chitosan-hyaluronate hybrid gel was injected at the time of meniscectomy in 9 animals and two weeks after meniscectomy in another four animals. The contralateral knee served as control to either saline or hyaluronate (1% gel 200-microliters, produced by Savient Pharmaceuticals, Inc., East Brunswick, NJ, USA) was injected.

The rats were allowed unrestricted motion after the surgery and evaluated every six weeks under image intensification. The following parameters were evaluated: degree of medial joint space opening and unloaded joint space width. After 3 months, the animals were sacrificed, and histological examination was performed.

Animal knees were randomized after incapacitance testing (see below) demonstrated similar weight-bearing on both hindlimbs. In one knee, either saline (in 4 animals) or hyaluronate was injected (5 animals). In the contralateral knee, chitosan-hyaluronate mixture was injected. In another four animals, the injection was performed under general anesthesia one week after meniscectomy.

### 2.2. Chitosan-Hyaluronate Hybrid Gel

A proprietary novel chitosan-hyaluronic acid hybrid (CHH) from Chi2Gel Ltd. (http://www.chi2gel.com/) has been used. Briefly, a mixture of chitosans and oligochitin (FM80, DAC50, and oligochitin from Koyo chemicals Ltd., Japan) was solubilized in HCl 0.13N and titrated with sodium hydroxide to near pH 7 forming a stable colloidal viscous mixture. This was followed by an addition of hyaluronic acid (molecular weight of 3 million Dalton, Ferring Ltd.). The resultant solution is a homogeneous liquid solution at 4°C that transforms into a gel at physiological conditions, that is, 37°C and pH 7.4. Genipin (Challenge Bioproducts Co., Ltd., Taiwan), a natural cross-linker is added to the CHH solution, at 0.2% (w/v) just before injecting and accelerates the gelation.

### 2.3. Incapacitance Tester Evaluation

Incapacitance tester is a device allowing assessing changes in hind paw weight distribution between the right (osteoarthritic) and left (contralateral control) limbs. It has been utilized as an index of joint discomfort and may be useful for the discovery of novel pharmacologic agents in human OA [16]. The animals were assessed prior to surgery as well as 24 hours after surgery for the relative amount of weight bearing on either knee using a Linton Incapacitance meter (Linton Instrumentation, Norfolk, UK). The animals were ranked according to the difference between right limb and left limb weight bearing. The experimental versus control knees were then determined so that there was similar distribution of right-limbed versus left-limbed animals. This step is important in order to prevent bias related to animal “handedness.” Animals were examined again two weeks after injection of the intra-articular therapy. The animals were examined again 14 days after instillation of the intra-articular therapeutic agent.

### 2.4. Histological Evaluation

 The rats were euthanized using intraperitoneal 200 mg/kg sodium pentobarbital injection. The knees were dissected out and processed for routine histology following fixation with 1% cetylpyridinium chloride—4% formalin solution for 48 hours. Decalcification was carried out in EDTA for three weeks on average. Masson's trichrome and hematoxylin stains were evaluated. The following parameters were measured: cartilage thickness at the lowest part of the medial femoral condyle, osteophyte formation, cyst formation, and subchondral bone plate thickness.

### 2.5. Statistical Analysis and Image Analysis

Quantitative histology was performed using an image analysis program (ImageJ [17]). Statistical analysis was performed using the Microsoft Excel add-in program Analyze-it version 2.22 [18].

## 3. Results

### 3.1. Animals

All animals survived the surgery, and their joint did not exhibit any evidence of inflammation or wound breakdown. Weight gain proceeded as expected with the animals gaining on average 100 grams during the follow-up period.

### 3.2. Incapacitance Tester Evaluation

The difference between the experimental and control knee averaged  1 ± 2  grams prior to surgery. The relative weight-bearing did not significantly change following meniscectomy (2 ± 2). After 2 weeks, the amount of weight bearing was measured again. The animals bore weight preferentially on the experimental knee (16.6 ± 4 grams). This difference was found to be significantly different from that measured 24 hours following surgery (Student's *t*-test, *P* < 0.017).

### 3.3. Histological Evaluation

**Figure 1:**
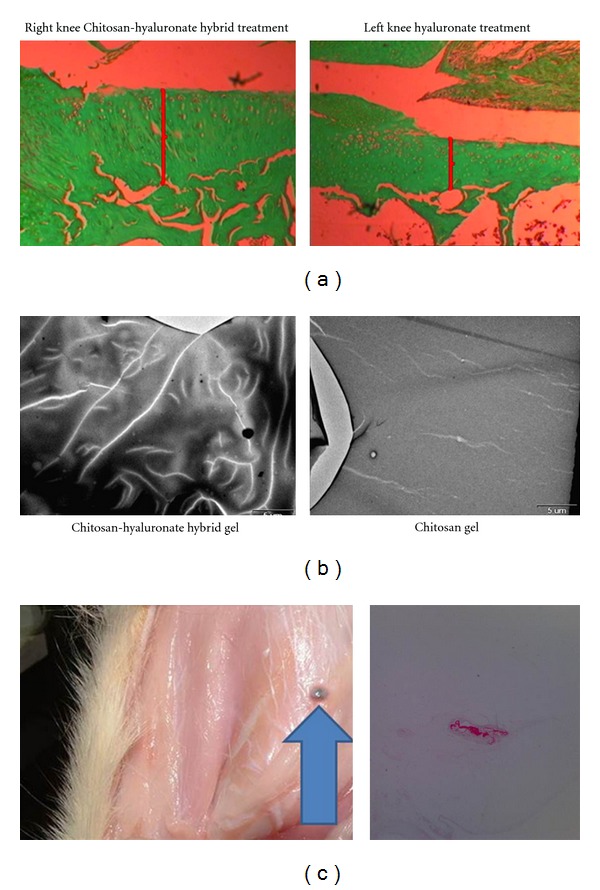
(a) rat knees following medial meniscectomy. Cartilage thickness is higher in the chitosan-hyaluronate-hybrid gel-treated knee than in the hyaluronate-treated knee. (b) environmental scanning electron microscopy seems to indicate that the hybrid gel has an internal structure. The authors hypothesize that the larger hyaluronate molecules (bright lines) appear to chaperon and organize the smaller chitosan molecules. (c) subcutaneous injection in rats does not evoke an inflammatory response macroscopically. The gel forms a discrete nodule (arrow head). This contrasts with the often observed intense inflammatory reaction previously reported with chitosan injection. The difference seems to be related to the method of preparation of the gel and its specific components. Histologically, the gel nodule (red) is surrounded by minimal fibrous capsule without inflammatory cells aggregation (original magnification ×10, Safranin red stain).

Four parameters were assessed by a blinded examiner.Cartilage thickness was increased in the experimental groups (170 ± 8) as compared with the control knees (108 ± 10) (Figure 1(a)). The difference was found to be significant (Student's *t*-test, *P* < 0.043). The hybrid gel appears to undergo self-assembly perhaps due to hyaluronate molecules aligning the smaller chitosan molecules (Figure 1(b)) and does not seem to induce an inflammatory response when injected subcutaneously in rats (Figure 1(c)).

Cyst grading was performed using a 4-point scale—0: no cyst, 1: minimal cyst, 2: large cyst, and 3: very large cyst. There were no cysts formed in the experimental group (average grade 0), while in the control group the average was 0.55 ± 0.5. This difference was found to be significant (Student's *t*-test, *P* < 0.047).

Subchondral bone plate thickness: results showed no significant difference between the groups.

Osteophyte grading was performed using a 4-point scale—0: no osteophyte, 1: minimal osteophyte, 2: large soft tissue osteophyte, and 3: large bony osteophyte. Average grade in the chitosan group was 0.8 ± 0.5, while in the control group it was 1.2 ± 0.3. This difference was not significant.

## 4. Discussion

Chitosan is a positively charged polymer and is biocompatible, non-toxic, and nonimmunogenic, allowing its use in the medical, pharmaceutical, cosmetic, and tissue reconstruction fields [19]. It has previously been shown to act as a coagulation agent in penetrating injuries [20]. The use of injectable chitosan has been limited to date due to its potential to cause neutrophil recruitment with inflammation-like effect and indeed prevents surgically induced immunosuppression [21]. Early work on chitosan back to 1999 demonstrated that intra-articular injection led to cartilage overgrowth and arthrofibrosis [22]. This was possible due to macrophage reaction observed when chitosan is degraded.

However, in recent years several methods of bypassing this problem were developed. Injection of mesenchymal cells embedded in a chitosan matrix allows intra-articular survival of the implanted mesenchymal cells, though these cells do not seem to have participated in cartilage reconstruction [23]. Indeed the use of intra-articular injection of chitosan appears to allow adhesion prevention following patellar fracture fixation (Chinese language article [24]). In addition, chitosan has been shown to improve joint lubrication when injected intra-articularly in humans (Chinese language article [24]). Chitosan has also been used together with a radioactive agent as a chemical synovectomy agent in humans [1] in phase I/IIa trials.

The current study demonstrates that the use of chitosan-HA hybrid injection delays osteoarthritis progression in a rat meniscectomy model. The injection of chitosan hybrid appears to be superior to either saline or hyaluronate injection. The possible mechanisms of action include adherence to cartilage as described for osteochondral cartilage defects [12] or a direct cartilage proliferation-enhancing effect as previously described by Lu et al. [22]. The results of this study concur with results obtained in previous studies demonstrating prevention of disc degeneration in rabbits as well as improved repair of rotator cuff tears in rats using a similar chitosan hybrid gel [25]. It is possible that the hyaluronate acts to mitigate the inflammatory effect observed when chitosan is degraded, thus explaining the better weight-bearing and histological features observed in this study.

The protective effect apparently leads to reduced knee pain as determined by increased weight-bearing on the chitosan-hybrid-injected knee as compared with the control knee.

In summary, it appears that the use of chitosan hybrid intra-articularly is possible, and that at least in an animal model it might delay osteoarthritis progression and improve knee function. Further studies are required to define the optimal timing of knee injections as well as the possible of repeated administration of the therapeutic agent. Further studies, including a large animal model, are needed in order to better assess whether such a biomaterial might prove beneficial in humans.

## References

[1] Song J, Suh CH, Park YB (2001). A phase I/IIa study on intra-articular injection of holmium-166-chitosan complex for the treatment of knee synovitis of rheumatoid arthritis. *European Journal of Nuclear Medicine*.

[2] Malette WG, Quigley HJ, Gaines RD (1983). Chitosan: a new hemostatic. *Annals of Thoracic Surgery*.

[3] Minami S, Okamoto Y, Hamada K, Fukumoto Y, Shigemasa Y (1999). Veterinary practice with chitin and chitosan. *EXS*.

[4] Hombach J, Bernkop-Schnürch A (2009). Chitosan solutions and particles: evaluation of their permeation enhancing potential on MDCK cells used as blood brain barrier model. *International Journal of Pharmaceutics*.

[5] Cho BC, Park JW, Baik BS, Kwon IC, Kim IS (2002). The role of hyaluronic acid, chitosan, and calcium sulfate and their combined effect on early bony consolidation in distraction osteogenesis of a canine model. *Journal of Craniofacial Surgery*.

[6] Liggins RT, Cruz T, Min W, Liang L, Hunter WL, Burt HM (2004). Intra-articular treatment of arthritis with microsphere formulations of paclitaxel: biocompatibility and efficacy determinations in rabbits. *Inflammation Research*.

[7] Liu SQ, Qiu B, Chen LY, Peng H, Du YM (2005). The effects of carboxymethylated chitosan on metalloproteinase-1, -3 and tissue inhibitor of metalloproteinase-1 gene expression in cartilage of experimental osteoarthritis. *Rheumatology International*.

[8] Thakkar H, Sharma RK, Mishra AK, Chuttani K, Murthy RSR (2004). Celecoxib incorporated chitosan microspheres: in vitro and in vivo evaluation. *Journal of Drug Targeting*.

[9] Usami Y, Okamoto Y, Minami S (1994). Migration of canine neutrophils to chitin and chitosan. *The Journal of veterinary medical science / the Japanese Society of Veterinary Science*.

[10] Ueno H, Murakami M, Okumura M, Kadosawa T, Uede T, Fujinaga T (2001). Chitosan accelerates the production of osteopontin from polymorphonuclear leukocytes. *Biomaterials*.

[11] Zhao R, Ren Y, Sun B, Zhang R, Liang D (2010). Experimental study on chitosan mediated insulin-like growth factor gene transfection repairing injured articular cartilage in rabbits. *Zhongguo Xiu Fu Chong Jian Wai Ke Za Zhi*.

[12] Marchand C, Chen G, Tran-Khanh N (2012). Microdrilled cartilage defects treated with thrombin-solidified chitosan/blood implant regenerate a more hyaline, stable, and structurally integrated osteochondral unit compared to drilled controls. *Tissue Engineering A*.

[13] Jingcheng W, Lianqi Y, Yu S (2012). A comparative study of the preventive effects of mitomycin C and chitosan on intraarticular adhesion after knee surgery in rabbits. *Cell Biochemistry and Biophysics*.

[14] Bendele A, Mccomb J, Gould T (1999). Animal models of arthritis: relevance to human disease. *Toxicologic Pathology*.

[15] Bethesda Animal Care and Use Committee NCI Rodent Anesthesia Protocols.

[16] Kobayashi K, Imaizumi R, Sumichika H (2003). Sodium iodoacetate-induced experimental osteoarthritis and associated pain model in rats. *Journal of Veterinary Medical Science*.

[17] NIH Image. Image Analysis IMAGE J.

[18] Analyse-it Software L. Analyze-it.

[19] Ben-Shalom N, Nevo Z, Patchornik A, Robinson D Novel injectable chitosan mixtures forming hydrogels.

[20] Azargoon H, Williams BJ, Solomon ES, Kessler HP, He J, Spears R (2011). Assessment of hemostatic efficacy and osseous wound healing using HemCon dental dressing. *Journal of Endodontics*.

[21] Kosaka T, Kaneko Y, Nakada Y, Matsuura M, Tanaka S (1996). Effect of chitosan implantation on activation of canine macrophages and polymorphonuclear cells after surgical stress. *Journal of Veterinary Medical Science*.

[22] Xi Lu J, Prudhommeaux F, Meunier A, Sedel L, Guillemin G (1999). Effects of chitosan on rat knee cartilages. *Biomaterials*.

[23] Jing XH, Yang L, Duan XJ (2008). In vivo MR imaging tracking of magnetic iron oxide nanoparticle labeled, engineered, autologous bone marrow mesenchymal stem cells following intra-articular injection. *Joint Bone Spine*.

[24] Chen AM, Hou C (2000). Clinical study of chitosan for Prevention of Knee Adhesion. *Academic Journal of Second Military Medical University*.

[25] Robinson D, Pachornik S, Shalom NB, Sagiv S, Melamed E, Nevo Z The use of a chitosan-based hyaluronate gel in musculoskeletal afflictions.

